# Gastrointestinal and respiratory morbidity when introducing eggs as complementary food: a randomised controlled trial in South African infants

**DOI:** 10.1038/s41598-024-76169-4

**Published:** 2024-10-29

**Authors:** Regina Nakiranda, Linda Malan, Hannah Ricci, Herculina S. Kruger, Arista Nienaber, Marina Visser, Cristian Ricci, Mieke Faber, Cornelius M. Smuts

**Affiliations:** 1 North-West University (Centre of Excellence for Nutrition), Potchefstroom, South Africa; 2 Africa Unit for Transdisciplinary Health Research (AUTHeR), North-West University, Potchefstroom, South Africa; 3 South African Medical Research Council (Non-Communicable Diseases Research Unit), Tygerberg, South Africa

**Keywords:** Nutrition, Gastroenteritis, Respiratory signs and symptoms

## Abstract

We investigated the incidence and duration of morbidity symptoms among infants aged 6 to 9 months from a low socioeconomic community receiving one egg daily for 6 months. This was a secondary outcome of a randomised controlled trial (RCT) of 500 infants conducted in Jouberton, South Africa. The primary outcome was linear growth. Morbidity data were collected weekly using a symptoms diary and qualitative data with focus group discussions at the endpoint. Ethical approval was obtained from the North-West University Health Research Ethics Committee. The intervention group had a ~ 5% higher incidence of gastrointestinal morbidity (17.0%) compared to the control group (11.9%). Gastrointestinal morbidity without fever tended to be 1.4 times higher in the intervention group (OR: 1.43, 95% CI: 1.03, 1.93; *P* = 0.058) and tended to be 4 times higher with fever (OR: 4.07, 95% CI: 0.86, 19.23; *P* = 0.077). The duration of total gastrointestinal and respiratory morbidity was 1.5 days longer in the intervention group (β: 1.491; 95% CI 0.064, 2.918; *P* = 0.041). Complementary feeding with eggs may have contributed towards an increased risk for gastrointestinal morbidity.

**Supplementary Information:**

The online version contains supplementary material available at 10.1038/s41598-024-76169-4.

## Introduction

Diarrhoea and acute respiratory infections continue to be major causes of morbidity and mortality in children under the age of five years, particularly in Sub-Saharan Africa^1^. In South Africa, diarrhoea is the third major cause of illness and death accounting for 20% of deaths and lower respiratory infections are the fourth leading cause of death in children under five years according to the second South African National Burden of Disease Study (SANBDS-2)^2^. Infections and undernutrition have a bidirectional relationship. Gastrointestinal infections with diarrhoea lead to malnutrition through the reduction of nutrient intake, increased metabolism and malabsorption, whereas undernutrition increases vulnerability to gastrointestinal and respiratory infectious diseases because of a weakened immune system^3,4^. Undernutrition is a major global public health problem accounting for almost half of deaths in under-five-year old children^5^. In South Africa, undernutrition remains a significant issue with a stunting rate of 27.4% among children under five years^6^. Therefore, adequate infant nutrition, including early breastfeeding initiation and exclusive breastfeeding for six months, together with appropriate complementary feeding (CF) practices are recommended. These practices are protective against gastrointestinal illnesses and respiratory illnesses^7–9^.

Complementary feeding is a crucial phase when infants transition to consuming other foods in addition to breast milk or formula milk, to meet their energy and nutrient requirements beyond what milk alone can provide^5^. Eggs are rich in essential macro and micronutrients required for infant nutrition^10^. Regular inclusion of eggs in complementary diets can serve as an affordable and sustainable nutritional intervention, particularly in impoverished communities, with the potential to enhance linear growth in infants^11^. Eggs also contain antimicrobial proteins, immune factors, and micronutrients such as vitamin A^10^. Vitamin A is an important factor in immune function, particularly in the reduction of gastrointestinal morbidity^12^. In South Africa, routine periodic high-dose vitamin A supplementation is performed according to WHO guidelines, but implementation during the Covid-19 pandemic during which this study was conducted, was uncertain.

The introduction of CF brings potential challenges, as diarrhoea and respiratory illness peak between 6 and 23 months. These illnesses can arise due to factors such as the displacement of protective breast milk by CF, unhygienic preparation practices, contamination of feeding utensils, and improper storage of feeds^8,13–16^. In addition, Environmental enteric dysfunction (EED) is prevalent in children living in low and middle-income countries, with frequent exposure to faecal pathogens^17^ and may be a cause of impaired gut permeability, malabsorption and inflammation^18,19^. It is associated with both poor growth and gastrointestinal morbidity in infants^19–21^.

Other causes of gastrointestinal morbidity during CF include food allergies, sensitivities or intolerances^22^ Data on food allergies in low and middle-income countries is limited due to the under-recognition of food allergies^23,24^. A South African food allergy study involving 1200 black children aged 12–36 months reported food sensitisation rates of 9.0% in urban areas and 1–2% had an egg allergy, especially to raw eggs^25^. Food intolerances affect 15–20% of the population depending on the method of data collection^26^.

According to a systematic review, studies examining CF interventions using eggs and other animal-based foods yielded mixed results regarding their effect on morbidity symptoms in infants^27^. A Cochrane review found low evidence of the effect of such CF interventions on all-cause morbidity due to inconsistencies in the studies and a lack of accuracy in the measurement of morbidity^28^. The Lulun study, a randomised controlled trial (RCT) conducted in Ecuador demonstrated a significant increase in episodes of diarrhoea among infants who consumed eggs daily as a complementary food^29^.

In addition, a qualitative study reported that caregivers’ cultural beliefs and attitudes influence the prevalence of reported diarrhoea in infants receiving CF^30^. In Ecuador, the higher prevalence of diarrhoea among daily egg consumers was attributed to biases arising from caregivers’ cultural beliefs and attitudes associated with eggs and diarrhoeal illnesses^30^.

The primary objective of the current RCT (Eggcel-growth) was to investigate the effect of providing one chicken egg per day for 6 months to infants aged 6 to 9 months on linear growth, which found no effect^31^. Here, we report on a secondary objective, the incidence and duration of gastrointestinal and respiratory morbidity among infants during the 6-month intervention when introducing an egg per day as CF compared to the usual habitual intake. A qualitative component was used to explore mothers/caregivers’ perceptions of morbidity symptoms, especially diarrhoea and vomiting and cultural beliefs about egg consumption and diarrhoea in infants.

## Materials and methods

### Study setting and population

The details of the Eggcel-growth design, methods, and primary outcomes were described elsewhere^31^. Briefly, infants between the ages of 6 to 9 months, whose mothers/caregivers provided consent, were recruited in the peri-urban area of Jouberton, located within the greater Matlosana Municipality in Klerksdorp, South Africa. Infants with severe illnesses requiring hospitalisation, severe malnutrition (WLZ < − 3), severe anaemia (Hb < 7 g/dl), congenital abnormalities, or known allergies/sensitivities to eggs were excluded from participation. Additionally, mothers/caregivers/legal guardians under the age of 18 years or planning to relocate from the study area were also excluded. Trained field workers identified potentially eligible participants from households within the study area and provided them with information about the study. Interested participants were then invited to the central study site, where mothers/caregivers were provided with detailed information and requested to give informed consent. The study was conducted from 16th February 2021 to 21st December 2021 within the COVID-19 period. All COVID-19 protocols were strictly followed in accordance with government regulations throughout the study period. The trial ended in December after exiting all participants from the study.

### Study design and intervention

Assessment of gastrointestinal and respiratory morbidity was a secondary outcome of the RCT with a parallel design to investigate the efficacy of providing one chicken egg per day for 6 months to infants from age 6 to 9 months from a low socioeconomic community in on linear growth. A qualitative component was added to assess perceptions of morbidity symptoms in the context of the current study and eggs as complementary feeding. Eligible infants were randomly assigned in a 1:1 ratio using a randomization sequence generated by the RANNOR function in SAS software version 9.4. A dataset of 500 tags (250 for each group) was created. These tags were combined with a sequence of pseudo-random numbers from a normally distributed variable. The random numbers were sorted to produce a random list of codes and tags, which were then used by research assistant to assign 250 participants to each group.

The intervention group received one chicken egg (about 50 g) per day for 6 months and the control group did not receive eggs. The chicken eggs used in the study were obtained from a single supplier and were handled and packaged in accordance with the regulations outlined by the South African Department of Agriculture, Forestry, and Fisheries^32^. Every family in the intervention group received 7 eggs per week for the intervention child, and an extra 5 eggs to share among the rest of the family members. Mothers were encouraged to adhere to the daily provision of eggs to the intervention child and were counselled not to share the child’s intervention products. Fieldworkers monitored the mothers/caregivers from both groups weekly and provided the intervention products to the egg group. The family of the control group received an incentive of 5 kg maizemeal monthly during the intervention and four dozen eggs at the completion of the study for participation in the study. Fieldworkers collected weekly morbidity questionnaires from both groups and compliance forms from the intervention group. Mothers/caregivers were educated about the importance of maintaining hygiene during egg preparation, providing the egg well cooked, and the different cooking methods. The eggs were either mixed with the infants’ preferred foods or given directly without any accompanying food. Blinding was done to the study site staff who collected data on anthropometric measurements and dietary intake, laboratory staff, and data analysts. However, the participants and fieldworkers were not blinded due to the nature of the intervention.

Both the intervention and control groups had equal access to primary healthcare services offered at local clinics, including child vaccinations and vitamin A supplementation. Allergic sensitisation to eggs was assessed by skin prick testing and allergic reactions were tested with a food challenge test (not in sensitised children) at enrolment and excluded from the study if positive. Mothers were educated on the identification of a possible egg allergic reaction. Enrolled participants suspected of having a reaction to eggs during the study were closely monitored and subjected to a skin prick test to assess sensitisation.

### Sample size

The Eggcel-growth study sample size powered for growth was described in detail elsewhere^31^. In a previous RCT in Ecuador, it was reported that 26% and 15% of children in the intervention group and control group developed diarrhoea, respectively^11^. The sample size of 500 participants in the Eggcel-growth study, using the same morbidity outcomes described above would result in a statistical power of 86% (82% considering up to 10% lost to follow-up). The power provided by the sample size for morbidity outcomes was calculated using the Web Power package of the R software version 4.0.2.

### Breastfeeding and dietary intake

Breastfeeding practices and usual consumption of foods for both groups were assessed for the week prior to baseline, midpoint, and endpoint using a structured unquantified food frequency questionnaire (FFQ) previously used in this setting^33^. Mothers from both groups were encouraged to continue breastfeeding and maintain the infants’ normal complementary diets as before the study.

Egg intake was neither promoted nor prohibited in the control group. An egg intake estimation was therefore calculated for all study participants regardless of group, to further explore associations of actual egg intake with morbidity in the total sample. A weekly FFQ conducted in both groups was used. It had four options: Every day, most days (4–6 days), once a week (1–3 days) and never. The options were scored as never = 0; 1–3 days = 2; 4–6 days = 5; and every day = 7; which was then multiplied by the number of eggs eaten at a time (usually one egg, but also ½ or 2 eggs) and by the number of times eaten per day (usually once or twice).

### Sociodemographic and anthropometric outcomes

Infant age, sex, birth weight, and gestational age at birth were transcribed from the health booklet. Maternal sociodemographic characteristics were collected at baseline using questionnaires which have been previously used in the study setting^33^. Infant weight, length, mid-upper arm circumference, and head circumference were measured at 3 time points, at enrolment (baseline), 3 months after enrolment (midpoint) and 6 months after enrolment (endpoint). The weight-for-age Z (WAZ), length-for-age Z (LAZ), weight-for-length Z (WLZ), Body mass index for age Z (BMIZ), mid-upper arm circumference-for-age Z (MUACZ), and head circumference-for-age, Z (HCZ) scores were calculated using World Health Organization’s (WHO) child growth standards specific for age and sex^34^.

### Morbidity outcomes

Mothers were provided with a visual wellness/illness diary to record their child’s daily symptoms, such as cough, fever, diarrhoea, skin rashes, and any clinic visits and medications administered. The rationale for using a diary was that it provided a clear description of the child’s illness and treatment history and could report multiple symptoms a child had at a given time without relying on the mother’s memory as is the case when using only retrospective questionnaires^35^. Fieldworkers collected this information during weekly home visits, verified it and trained nurses classified the symptoms into gastrointestinal and respiratory morbidity before data was captured, as previously done in another study^33^. Incidences of morbidity symptoms were separated by 2 days without the symptoms^36^. Diarrhoea was defined as three or more watery stools in a day. Diarrhoea with or without vomiting plus fever was classified as gastrointestinal morbidity with a fever and without fever as gastrointestinal morbidity without fever. Cough (wet/dry) with or without runny/blocked nose, with or without fast/difficult breathing or wheezing plus fever was classified as respiratory morbidity with fever, and without fever as respiratory morbidity without fever. Longer durations of morbidity were defined as gastrointestinal morbidity longer than 14 days (or persistent diarrhoea) according to WHO^37^ and respiratory morbidity longer than 4 weeks (28 days) according to the 2020 Chest Consensus Statement^38^.

Information regarding vitamin A supplementation, HIV exposure, and chronic medications was obtained from the infants’ primary healthcare Road-to-Health booklet. Infants who experienced severe adverse events, severe illness, or any concerning conditions identified through the data (e.g., illness lasting longer than 14 days) were referred to the Department of Paediatrics at Klerksdorp Hospital for further management.

### Biochemical measurements

At baseline and endpoint, a trained nurse collected a capillary blood sample of approximately 350 µL from a finger or heel prick. The blood was collected in lithium heparin tubes, centrifuged for 10 min at 2000 g, and plasma was aliquoted into 0.2 ml Eppendorf tubes. The aliquots were frozen at -20 °C on-site and subsequently stored at -80 °C until analysis. Analysis of retinol-binding protein (RBP), soluble transferrin receptor [sTfR]) and inflammatory markers (C-reactive protein [CRP] and alpha-1 acid glycoprotein [AGP]), and Environmental Enteric Dysfunction (EED) markers including intestinal fatty acid–binding protein (I-FABP), soluble CD14 (sCD14), and markers of growth hormone resistance including insulin-like growth factor 1 (IGF-1), and fibroblast growth factor 21 (FGF21) was conducted using the Q-Plex Human Environmental Enteric Dysfunction (11-Plex) TM assay (Quansys Bioscience, Utah, USA)^39^. The results were interpreted using the Q-View™ software.

### Qualitative data collection

A qualitative study was added to the current RCT to explore mothers/caregivers’ perceptions of morbidity symptoms, especially diarrhoea and vomiting and cultural beliefs about egg consumption and diarrhoea in infants. A qualitative descriptive design^40^ was used and purposive sampling was used to select participants for the focus group discussions (FGDs). Participants were selected at the midpoint study visit based on maternal age, since maternal experiences may vary between young mothers and older mothers. An equal number of participants were selected from the intervention group (10) and control group (10). After providing additional informed consent for the FGDs, three FGDs with at least 6 people per group were conducted towards the end of the study. One group consisted of young mothers of 18–24 years (4-intervention and 3-control), another group was of older mothers of > 25 years (3-intervention, and 3-control), and the third group was mixed (3-intervention and 4-control) and all the mothers who participated had already exited the study. A focus group discussion guide was used. One person was the facilitator, one took notes, two people made general observations and an audio recorder was used. The focus group discussion was conducted mostly in Setswana (the main local language spoken in this area) and English. All COVID-19 protocols were observed. Data saturation was achieved as we could not get any new responses from the groups after the second FGD and previous responses were repeated during the third FGD.

FGD data was transcribed verbatim. Two independent researchers identified codes from the transcribed data, discussed the codes and reached a consensus on the extracted codes. Codes were grouped according to categories and categories were grouped according to themes to present the results. We used content deductive thematic analysis as described by Caufield^41^. Direct quotations from the respondents were used in the presentation of study findings according to themes^42^. Creswell’s method was used to ensure the validity of the data by triangulation, using thick descriptions of data, including negative information in the themes^43^.

### Statistical methods

Baseline characteristics were described by treatment allocations as counts and percentages for categorical data and medians and interquartile range (IQR) for skewed continuous data or mean and standard deviation (SD) for normally distributed continuous data. Incidence and duration of gastrointestinal and respiratory morbidity were analysed by generalised linear mixed models with random intercept, using treatment group for assessing effects of the intervention, and association with the egg intake estimation regardless of group allocation, respectively, as exposure variables adjusting for age, sex, stunting rate, baseline breastfeeding, HIV exposure, maternal marital status, maternal education level, low birth weight, baseline FGF21, and baseline IDE. A likelihood-based ignorable analysis was applied for missing values^44^. The egg dose-response curve was performed using a restricted cubic spline with four knots placed at the 5th, 35th, 65th and 95th percentiles of egg intake estimation. Differences in RBP concentrations between intervention and control groups were assessed by independent student t-test. The effect of egg consumption on vitamin A status was assessed by ANCOVA adjusting for baseline RBP, and vitamin A supplementation at the endpoint. Association between foods consumed, vitamin A status, vitamin A supplementation, and study treatment (control or intervention) was assessed by Chi-Square test. RBP and TfR was corrected for inflammation using the Biomarkers Reflecting Inflammation and Nutritional Determinants of Anaemia (BRINDA) regression for preschool-aged children^45^. A *P* ≤ 0.05 was considered significant for all inferential analysis. The statistical analyses were performed using SAS vers. 9.04.

### Ethical approval

The study followed the guidelines laid down in the Declaration of Helsinki. Ethical approval was obtained from the Health Research Ethics Committee of the North-West University (NWU-00452-19-A1) and the study was registered on clinicaltrials.gov (NCT05168085) on 13/02/2020. Goodwill permission was sought from the South African National and Provincial Department of Health, the local clinics in the study area and the community leaders to do the study. Written informed consent was obtained from all parents/legal guardians.

## Results

**Figure 1:**
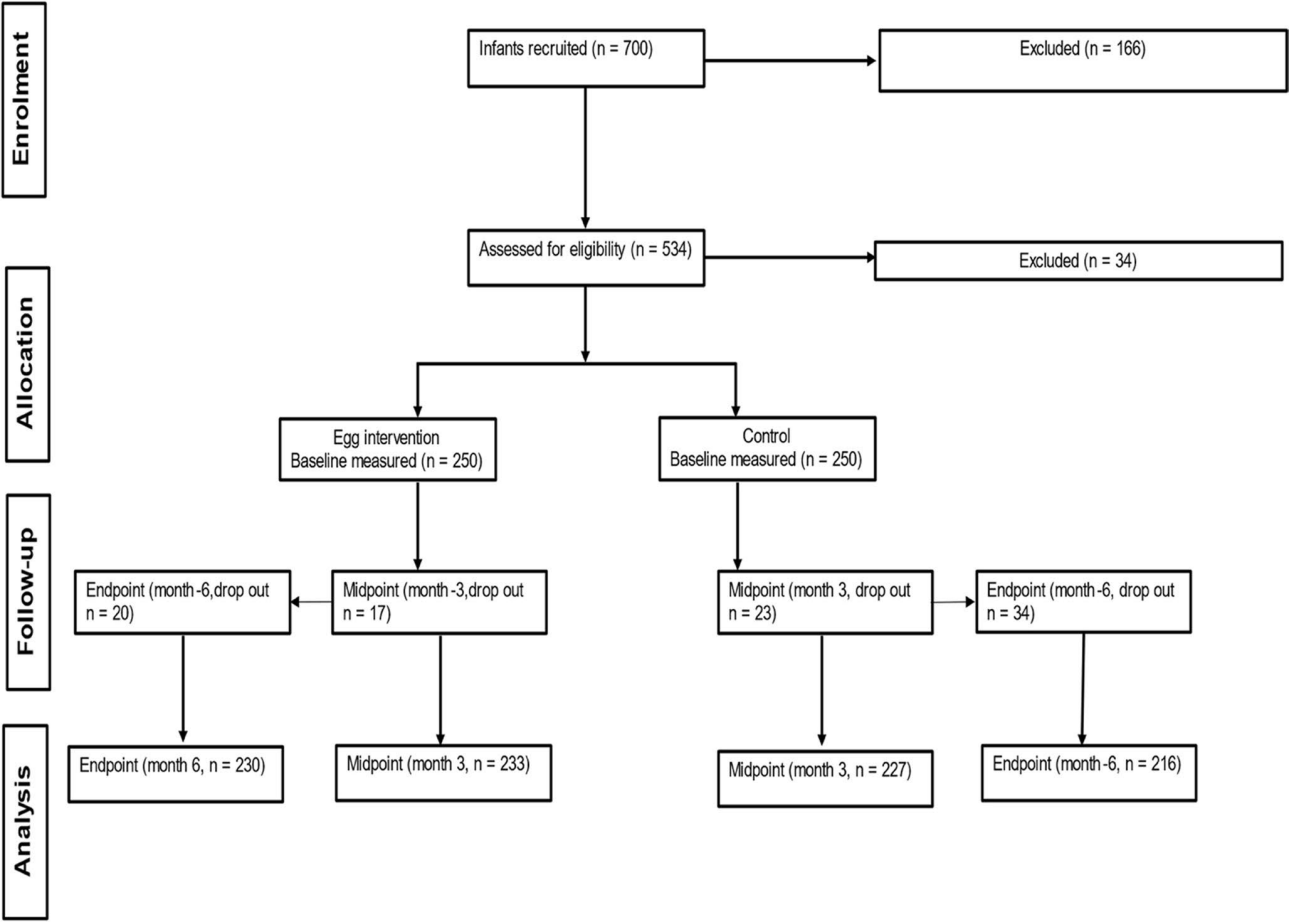
Flow diagram of participant progression through the randomised control trial.

Out of 500 enrolled participants, 54 infants (10.8%) dropped out of the study (Fig. 1). Table 1 shows the baseline characteristics of study participants. At enrolment, the median age of infants in the intervention group was 6.5 months, while in the control group, it was 6.6 months. Similar proportions of the infants from the intervention (30%) and control groups (31.2%) were exposed to HIV. Baseline elevated CRP was 15% and 7.9% in the intervention and control groups respectively. Of the 446 (89.2%) infants that completed the study, 81 (16.2%) reported no illness events throughout the study.

**Table 1:** Baseline characteristics of 6 to 9-month-old infants by intervention group.

	Intervention group (*n* = 250)	Control group (*n* = 250)
Infant characteristics		
Age, months	6.46 (6.11, 7.66)^**†**^	6.57 (6.14, 7.52)
Girls n (%)	133 (53.2)	125 (50.0)
HIV-exposure n (%)	75 (30.0)	78 (31.2)
Gestational age, weeks	39 (38, 40)	39 (38, 40)
Low birth weight (< 2.5 kg), *n (%)*	38 (15.2)	46 (18.4)
Anthropometric status		
Weight, kg	7.60 (6.79, 8.31)	7.63 (6.93, 8.42)
Length, cm	65.0 (63.0, 67.05)	65.07 (63.30,67.0)
Mid-upper arm circumference, cm	14.85 (14.0, 15.65)	15.0 (13.85, 15.85)
Head circumference, cm	43.40 (42.30, 44.15)	43.23 (42.0, 44.40)
LAZ	-1.21 (-2.01, -0.55)	-1.13 (-1.87, -0.52)
WAZ	-0.31 (-1.31, 0.38)	-0.29 (-1.23, 0.56)
WLZ	0.51 (-0.15, 1.33)	-0.59 (-0.24, 1.39)
MUACZ	0.65 (-0.11, 1.32)	0.67 (-0.28, 1.47)
HCZ	0.02 (-0.66, 0.66)	-0.05 (-0.70, 0.61)
Stunting, n *(%)*	63 (25.2)	56 (22.4)
Underweight, *n (%)*	26 (10.4)	23 (9.2)
Wasting, *n (%)*	4 (1.6)	2 (0.8)
Overweight (WLZ > 2), *n (%)*	29 (11.6)	40 (16.0)
Micronutrient status		
Soluble transferrin receptor(sTfR), mg/l	9.73 (9·11,10.38)	10·21 9·54,10·93
IDE(sTfR > 8·3 mg/l) *n (%)*	107 (45.9)	89 (41.6)
Retinol binding protein (µmol/L)	1.795 ± 0.491^‡^	1.863 ± 0.552
VAD (RBP ≤ 0.7 µmol/L) *n (%)*	0 (0.0)	0 (0.0)
Vitamin A supplementation *n (%)*	137 (54.8)	146 (58.4)
Biochemical markers ^§^		
*Inflammatory status*,* n (%)*		
Inflamed, CRP > 5 g/L	35 (15.0)	17 (7.9)
Inflamed, AGP > 1 g/L	106 (45.5)	100 (46.7)
Environmental enteric dysfunction markers^¶^		
IGF-1 (ng/mL)	14.90 (6.69, 32.57)	18.47 (8.51, 31.74)
FGF21 (pg/mL)	165 (47, 480)	171 (43, 391)
I-FAB (pg/mL)	781 (546, 1180)	810 (600, 1177)
sCD14 (ng/mL)	2258 ± 711	2185 ± 594
Mother/caregiver characteristics		
Age, years	29 (23, 36)	27 (23, 33)
Education (≥ grade 10), *n (%)*	194 (77.6)	209 (83.6)
Married (incl. traditional), *n (%)*	34 (13.6)	22 (8.8)

^†^Median (25th, 75th ), all such values.

^‡^Mean ± SD, all such values.

^§^Corrected for inflammation using the Biomarkers Reflecting Inflammation and Nutritional Determinants of Anaemia (BRINDA) regression for preschool-aged children^45^.

^¶^ A total on *n* = 447analysed, *n* = 233 in intervention group and *n* = 214 in control group.

AGP, alpha-1 acid glycoprotein, CRP, C-reactive protein FGF21, fibroblast growth factor 21; HAZ, height/length-for-age; IDE,, iron deficiency erythropoiesis; I-FABP, intestinal fatty acid–binding protein; IGF-1, insulin-like growth factor 1; MUACZ, mid-upper arm circumference z-score; OR, odds ratio; sCD14, soluble CD14; VAD, vitamin A deficiency; WAZ, weigh-for-age z-score; WHZ, weight-for-height/length z-score.

### Incidence of gastrointestinal and respiratory morbidity

Table 2 shows the effect of the egg intervention on the incidence of gastrointestinal and respiratory morbidity during the 6 months. There was a ~ 5% higher rate of gastrointestinal morbidity in the intervention group (137/230 [17%]) compared to the control group (97/216 [11.9%]). The odds of having gastrointestinal morbidity without fever tended to be 43% higher in the intervention group (OR: 1.43, 95% CI: 1.03, 1.93; *P* = 0.058) and with fever tended to be 4 times higher in the intervention group (OR: 4.07, 95% CI: 0.86, 19.23; *P* = 0.077).

**Table 2:** Effect of egg intervention on incidence of gastrointestinal and respiratory morbidity during the 6-month intervention.

Outcome	Intervention group (*n* = 230) *n* (%)	Control group (*n* = 216) *n* (%)	OR (95% CI)	*P* value^*^
Gastrointestinal and respiratory morbidity	416 (51.5)	365 (44.8)	1.34 (1.04;1.74)	**0.026**
Gastrointestinal morbidity				
All	137 (17)	97 (11.9)	1.43 (1.03; 1.93)	**0.033**
With fever	8 (1.0)	2 (0.2)	4.07 (0.86; 19.23)	0.077
No fever	129 (16)	95 (11.7)	1.38 (0.99; 1.91)	0.058
Respiratory morbidity				
All	298 (36.9)	284 (34.9)	1.13 (0.86; 1.49)	0.375
With fever	53 (6.6)	51 (6.3)	1.07 (0.69;0.1.67)	0.759
No fever	245 (30.4)	236 (29)	1.09 (0.83; 1.44)	0.529

* Difference in incidence of gastroenteritis and respiratory morbidity between groups was assessed using generalised linear mixed (GLM) models with random intercept with treatment group as the exposure variable, adjusted for age, sex, stunting rate, baseline breastfeeding, HIV exposure, maternal marital status, maternal education level, low birth weight, and baseline FGF21 and IDE. *P* ≤ 0.05 was considered significant.

**Figure 2:**
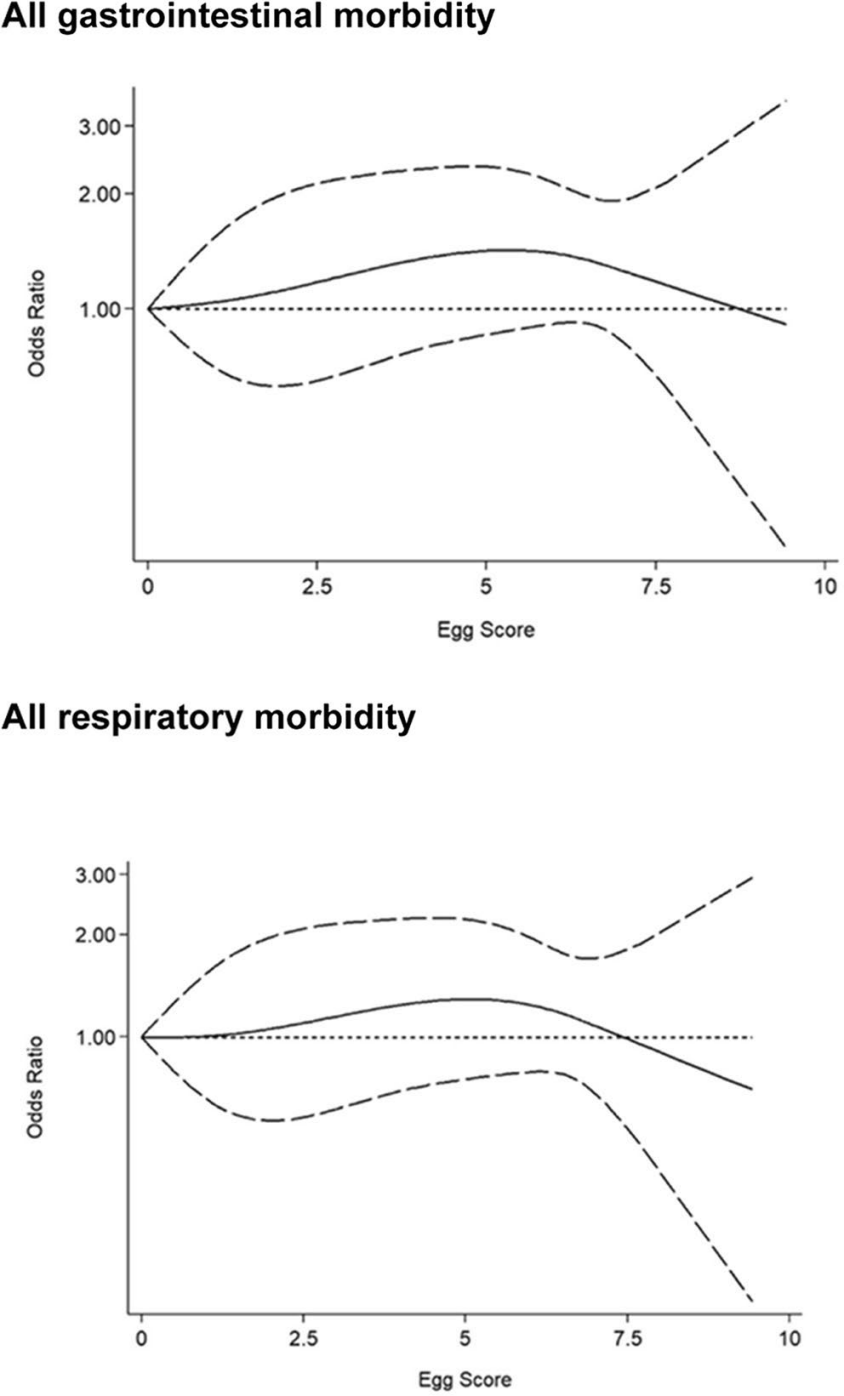
Egg dose-response of gastrointestinal and respiratory morbidity during the 6-month intervention. The dose-response analysis was adjusted for age, sex, baseline stunting, baseline breastfeeding, HIV exposure, marital status, education level, and baseline fibroblast growth factor 21 and iron deficiency erythropoiesis.

When considering estimated egg intake in both groups, there was no significant association between an estimated 1 egg increase weekly and the incidence of gastrointestinal and respiratory morbidity (OR:1.04, 95% CI 0.99; 1.09; *P* = 0.1613), after adjusting for age, sex, stunting rate, baseline breastfeeding, HIV exposure, maternal marital status, maternal education level, low birth weight, baseline FGF21, and baseline IDE (Table 3). In addition, there was no significant dose-response between 1 egg increase weekly and the risk of gastrointestinal or respiratory morbidity in the two groups (Fig. 2).

**Table 3:** Association of egg intake by 1 unit increase (1 egg increase weekly) with the incidence of gastrointestinal and respiratory morbidity during the 6-month intervention in the total sample irrespective of group allocation.

Outcome	OR (95% CI)	*P* value*
Gastrointestinal and respiratory morbidity	1.04 (0.99; 1.09)	0.1613
Gastrointestinal		
All	1.03 (0.97; 1.10)	0.3280
With fever	1.12 (0.87; 1.44)	0.3726
Without fever	1.03 (0.96; 1.09)	0.4182
Respiratory		
All	1.02 (0.97; 1.07)	0.5251
With fever	1.02 (0.94; 1.11)	0.6123
Without fever	1.00 (0.95; 1.06)	0.8738

*Association of egg intake by I unit (1 egg increase weekly) with the incidence of gastroenteritis and respiratory morbidity in the total sample was assessed using the GLM model with egg intake as the exposure variable, adjusted for age, sex, stunting rate, baseline breastfeeding, HIV exposure, marital status, education level, low birth weight, and baseline FGF21 and IDE. *P* ≤ 0.05 was considered significant.

### Duration of gastrointestinal and respiratory morbidity

Table 4 shows the effect of the egg intervention on the duration of gastrointestinal and respiratory morbidity during the 6-month intervention. The duration of total gastrointestinal and respiratory morbidity combined was 1.5 days longer in the intervention group compared to the control group (β: 1.491; 95% CI: 0.064, 2.918; *P* = 0.041). When excluding gastrointestinal morbidity longer than 14 days and respiratory illness longer than 28 days, gastrointestinal morbidity was ~ 6 h longer in the intervention group compared to control (β: 0.263; 95% CI: 0.047, 0.479; *P* = 0.017). Gastrointestinal morbidity with fever was ~ 1.2 h longer in the intervention group compared to control (β: 0.052; 95% CI: 0.005; 0.099; *P* = 0.030). Neither gastroenteritis nor respiratory morbidity duration differed between the two groups.

**Table 4:** Effect of egg intervention on the duration of gastrointestinal and respiratory morbidity compared to usual habitual intake (control) during the 6-month intervention.

Duration in days	Intervention group (*n* = 230)	Control group (*n* = 216)	β (95% CI)	*P* value^*^
Gastrointestinal and respiratory morbidity	6 (3, 11)^†^	6 (3, 10)	1.491 (0.064; 2.918)	**0.041**
	5 (3, 7)	5 (3, 7)	0.229 (-0.319; 0.778)^‡^	0.413
Gastrointestinal morbidity				
All	4 (3, 7)	4 (2, 6)	0.150 (-0.254; 0.553)	0.468
	4 (2, 6)	4 (2, 6)	0.263 (0.047; 0.479)^‡^	**0.017**
With fever	7 (4, 11)	2 (3, 3)^§^	0.012 (-0.136; 0.159)	0.875
	7 (3, 10)	2 (3, 3)^§^	0.052 (0.005; 0.099) ^‡^	**0.030**
Without fever	4 (2, 7)	4 (2, 6)	0.140 (-0.235; 0.514)	0.465
	4 (2, 6)	4 (2, 6)	0.213 (0.001; 0.425)^‡^	**0.050**
Respiratory morbidity				
All	7 (4,14)	7 (3,11)	1.250 (-0.190; 2.690)	0.089
	7 (4, 11)	7 (3, 10)	0.375 (-0.246; 0.997)^‡^	0.237
With fever	10 (6, 17)	7 (4, 11)	0.453 (-0.672; 1.578)	0.430
	9 (5, 14)	6 (4, 10	0.152 (-0.133; 0.437)^‡^	0.297
Without fever	7 (3, 14)	7 (3, 10)	0.837 (-0.319; 1.993)	0.157
	6 (3, 10)	7 (3, 10)	0.152 (-0.133; 0.437)^‡^	0.297

^†^Median duration (days) (25th, 75th ) for children who experienced morbidity, all such values.

*Effect egg intervention on the duration of gastroenteritis and respiratory morbidity was assessed using the GLM model in all children, with the treatment group as exposure variables, adjusted for age, sex, stunting rate, baseline breastfeeding, HIV exposure, maternal marital status, maternal education level, low birth weight and baseline FGF21 and IDE. ^‡^Excluding durations > 14 days and > 28 days for gastroenteritis and respiratory illness, respectively. *P* ≤ 0.05 was considered significant.

^§^Only two children in the control group had gastrointestinal morbidity with fever.

### Dietary intake

Supplementary Table S1 displays that the mean (5th, 95th percentile) estimated weekly egg intake of intervention group was significantly higher at 7.0 (4.0, 7.0) eggs than the control group at 0.5 (0.0, 5.0) during the 6-month study (*P* < 0.001). Supplementary Table S2 shows the foods usually consumed during the prior week at baseline, midpoint, and endpoint. Breastfeeding rates were similar in the intervention and control groups (60.4% versus 62.8% at baseline; 48.7% versus 51.9% at endpoint), as was the percentage of children who had formula milk feeds daily (50.8% versus 49.2% at baseline; 36.5% versus 33.6% at endpoint). At the endpoint, a higher proportion of children in the intervention group consumed milk (31.7% versus 22.2% in the control group, *P* = 0.024) and infant cereal (24.3% versus 16.7% in the control group, *P* = 0.045) at least 4 days/week. For cereals, roots and tubers, potato was the only food that differed between the two groups, although consumed by only a small percentage of children (9.6% in intervention vs. 15.7% in control group, *P* = 0.049 at endpoint). In terms of animal source foods, frequency of consumption of flesh foods (chicken, meat, liver and fish) is reported for at least 1 day/week. Chicken was the most consumed flesh food at all three time points, with no difference between the two groups. Meat was consumed by a higher percentage of children in the control group at midpoint (22.5% versus 15.0% in the intervention group, *P* = 0.041) and endpoint (39.9% versus 28.3% in the intervention group, *P* = 0.017). Egg intake (≥ 4 days/week) was similar in the two groups at baseline; but as expected, was significantly higher in the intervention group at both midpoint (88.0% versus 11.5%, *P* < 0001) and endpoint (82.8% versus 13.4% *P* < 0001). There was no difference in maize intake between groups.

### Vitamin A status and supplementation

Supplementary Table S3 shows vitamin A status and supplementation before and after the 6-month egg intervention. At baseline, all children in the study were vitamin A sufficient. Similar proportions of infants in the intervention (54.8%) and control groups (58.4%) had received one dose of vitamin A supplementation before the study, and this improved to 83.2% and 81.6% respectively at the end of the study. The egg intervention prevented a decline in RBP concentration in the intervention group compared to the control group (Beta = 0.469; 95% CI 0.393, 0.544; *P* < 0.001).

### Qualitative results

Table 5 shows the codes, categories, themes and textual citations of perceptions of FGD participants on diarrhoea and vomiting in the context of using eggs as a complementary food. We identified 5 themes from the FGDs related to diarrhoea and vomiting.

**Table 5:** Codes, categories, themes and textual citations of perceptions of FGD participants on diarrhoea and vomiting in the context of using eggs as complementary food.

Diarrhoea			
Code	Category	Themes	Textual citations
-Running stomach -Watery stool -Yellow stool	-Watery stool -Stool colour change	Recognition	*- Diarrhoea is when the baby has a running stomach-FGD1 P3.* *- When the baby’s stool is watery-FGD2 P2.* *- When the stool is changing colour to yellow-FGD2 P3.*
-Frequent stools -Cramps -No appetite	Excessive stool frequency (watery, smelly, frequent) Accompanying symptoms, weakness, poor appetite, and cramps)	Severity assessment	*- When the stool is liquid*,* and nappies are changed now and then I know it’s serious diarrhoea-FGD3 P1* *- When the child has diarrhoea*,* the nappies don’t smell good*,* that’s when I know that something is not right*,* and usually I change nappies now and then- FGD1 P2.*
-Poor hygiene -Infection (bacteria) -Seasonal changes -Developmental stage (teething, talking) Food-induced	-Environmental conditions -Developmental stage -Food allergy	Causative factors and food triggers	**-***When the bottles and baby dishes are not cleaned properly FGD3 P2* *- When the baby eats something from the floor with bacteria*,* it can make the baby have diarrhoea-FGD3 P2* *- Most kids have diarrhoea due to teething and when they start eating different foods-FGD1 P2* *- Food can cause diarrhoea and weather changes FGD2 P1*
**-**Eggs not a cause -Overeating eggs **-**Contaminated eggs benefits of eggs	-Eggs as triggers -Overeating eggs -Contaminated eggs	Egg related beliefs	**-***Yes*,* boiled eggs can give baby diarrhoea-FGD3 P1* *- If you feed the baby too much egg*,* the baby can have diarrhoea (laughs)-FGD3 P3* **-***I was told raw eggs boost the appetite*,* so I gave raw eggs to the baby to improve appetite-FGD1 P3* **-***Yes*,* rotten eggs can make babies have diarrhoea-FGD3 P5.* **-***No eggs don’t give the baby diarrhoea-FGD2 P1*
-Oral rehydration -Medications	-Oral rehydration -Medications	Management strategies	**-***I gave Motswako*,* I mixed 2 L of water*,* 8 teaspoons of sugar and ½ teaspoon of salt-FGD1 P2* **-***When the baby has too much diarrhoea*,* I make a mixture from ORS*,* which I got from the clinic*,* it’s for when the baby has lost a lot of water-FGD3 P2*
Vomiting			
-Expulsion of food from the stomach	-Expulsion of food from the stomach	Recognition	**-***Vomiting is when you can see that the baby throws up all the food you gave them-FGD1 P4* **-***When the food comes out forcefully-FGD3 P1.*
**-**Any vomiting -Frequent vomiting	-Frequency of vomiting	Severity assessment	**-***When I feed the baby once and they throw up and later I feed the baby again but if they throw up again then it’s serious P2* *- When every food and water you give the baby doesn’t settle in the stomach then the baby throws it up-FGD3 P1*
-Poor hygiene -Individual contact -Overeating -Dairy products	-Poor hygiene -Exposure to certain individuals -Overeating -Dairy products	Causes and Triggers	**-***When you don’t clean the breast before giving the baby breastmilk*,* I think the baby got an infection from breastfeeding*,* that’s why the baby was vomiting-FGD3 P5* *- When baby eats things from the ground he vomits-FGDG1 P2* **-***When I introduced my baby to a new food*,* it caused vomiting-FGD2 P1.* *- A person who has had an abortion is not allowed to hold the baby as it can cause the baby to vomit-FGD2 P2*
-Raw eggs -Egg smell	-Uncooked/half-cooked eggs -Egg smell	Egg related beliefs	**-***Yes*,* when the baby doesn’t want the egg*,* she can vomit it out-FGD3 P1* **-***Yes*,* when the eggs are not cooked properly P2* **-***When eggs are watery*,* they can make the baby vomit-FGD3 P4* ***-****No eggs don’t make the baby vomit-FGD1 P1*
-Oral rehydration -Raw eggs -Homemade remedies -Sweetened beverages Medical checkup	-Oral rehydration -Raw eggs -Non-medical homemade remedies -Sweetened beverages Medical checkup	Management strategies	**-***I took the baby to the clinic and she was referred to the hospital where she was given IV fluids*,* antibiotics*,* zinc pills*,* and Motswako-FGD3 P2* **-***I gave water and raw eggs-FGD1 P3* **-***I fry garlic and give the baby garlic oil*,* I use Xcella cooking oil (laughs*)-*FGD1 P5.* - *Coke*,* you shake it and take out the gas*,* then you give the baby-FGD2 P1* *-My grandmother told me black tea helps with vomiting-FGD1 P4*

### Recognition of symptoms

The FGD participants defined diarrhoea as watery or frequent stools, or if stool changes colour to yellow or green. Participants described vomiting as the expulsion of food or liquids from the stomach.

### Severity assessment

The FGD participants reported that serious diarrhoea is generally indicated by excessive stool frequency or accompanying symptoms like cramps or loss of appetite. Serious vomiting was described by mothers as frequent episodes.

### Causative factors and food triggers

The FGD participants believed that diarrhoea is caused by poor hygiene, developmental stages like teething, food, bacteria and seasonal changes. The mothers had varying opinions about the causes of vomiting, mentioning poor hygiene, overeating, and exposure to certain individuals as causes.

### Egg related beliefs

More than half of the FGD participants thought eggs do not cause diarrhoea, but some expressed concerns about excessive egg consumption causing diarrhoea. When asked about the use of raw eggs, one mother reported raw eggs are used to help reduce slime or give appetite to the baby. Three-quarters of the mothers indicated that eggs do not make babies vomit. However, some expressed concerns that if the baby is intolerant to the egg or if eggs are not cooked properly, they could make the baby vomit.

### Management strategies

Two-thirds of the mothers reported using remedies for diarrhoea or vomiting like rehydrating the infants with Motswako (a mixture of 2 L of water, 8 teaspoons of sugar and ½ teaspoon of salt) or Oral Rehydration Salts (ORS) or water and mentioned the use of medications. Mothers’ opinions on how to manage vomiting were varied but more than half of them mentioned rehydrating the child with water and sugar, in addition, a quarter of the mothers mentioned non-medical home treatment for diarrhoea or vomiting, like oil, black tea, or sweetened beverages. One participant mentioned the use of raw eggs to manage vomiting, and a few took their babies for medical checkups.

## Discussion

This RCT, among 6- to 9-month-old infants from a low socioeconomic community in South Africa showed that eggs may have increased the risk of gastrointestinal morbidity with and without fever and had no effect on respiratory illnesses. This is similar to a study in Ecuador which reported a higher incidence of acute diarrhoea of 26% in the intervention compared with 15% in the control group^11^.

Although a small proportion of ~ 1% of the children were affected by gastrointestinal morbidity with fever, the increase in the intervention group could be due to several possible factors. Firstly, it could have been due to food-borne illnesses (bacterial or viral) resulting from unhygienic food preparations, especially because gastrointestinal morbidity with fever tended to be four times higher in the egg group. As reported by Iannotti et al.^11^, this could have been due to improper egg preparation and handling e.g. the lack of handwashing, undercooked or raw egg consumption, and improper storage of complementary feeds mixed with eggs^14–16^. Additionally, cultural practices associated with eggs might have exposed the infants to food-borne illnesses increasing the risk of diarrhoea, e.g. the qualitative data revealed mothers use raw eggs to help reduce slime and give appetite to the baby. A study in 2017 in South Africa found that 73% of commercial egg samples had bacterial contamination^46^. Therefore, the children in the egg group could have been more exposed to S*almonella Enteritis* serova *Enteridis*, the main food-borne pathogen associated with egg consumption. Even though, *Salmonella Enteritis* is destroyed by cooking, it can persist in undercooked eggs^47^.

Secondly, it could be a result of over-reporting of the presence of diarrhoea in the intervention group and or under-reporting in the control group. This is supported by the fact that there was no significant association of the egg intake estimation with gastrointestinal morbidity nor a significant egg dose-response and gastrointestinal morbidity incidence. Reporting biases may have arisen from the inability to blind the intervention. In this study, the prevalence of diarrhoea was 17% in the intervention group, which is similar to the South African Demographic and Health Survey (SADHS) 2016 report, where the prevalence of diarrhoea two weeks prior to the survey was 16–17% among 6–23-month-old infants^6^. This suggests that there may have been underreporting of the diarrhoea in the control group (~ 12%).

Mothers’ cultural beliefs and perceptions have been previously highlighted in a qualitative study to influence the prevalence of diarrhoea^30^. In our study, some FGD participants worried that excessive egg consumption could cause diarrhoea and vomiting in infants intolerant to eggs. Similar concerns were noted in Ecuador, where increased diarrhoea was linked to caregivers’ beliefs about eggs causing diarrhoea due to their fat content^30^. An Ethiopian study also reported more vomiting in a group consuming one egg per day plus eggshell powder, though no difference in diarrhoea symptoms was observed^48^.

IgE-mediated egg allergy has also been reported as a cause of gastrointestinal morbidity in children consuming eggs^49^.In addition, children may have had undiagnosed non-IgE mediated egg allergy or intolerance to egg that can trigger digestive distress, including diarrhoea^50^. Although in our study, there was no difference in egg sensitisation in the intervention group in the control group, (unpublished data) and children with egg allergy and/or sensitisation were excluded from the study.

There was no effect of egg intervention on respiratory illnesses similar to the Ecuador study^11^. This is contrary to a study in Rural Cotopaxi, Ecuador where 6–9-month-old infants consuming eggs had a lower prevalence of respiratory symptoms after adjusting for sex, maternal unemployment, and undernutrition compared with those who did not consume eggs or dairy products^51^.

Even though 30.5% of the infants were HIV exposed, only one child was HIV infected and on ART owing to the Prevention of Mother to Child Transmission of HIV (PMTCT) programme in South Africa^52^. The HIV exposure status did not differ among groups and had no association with morbidity symptoms.

Primary care vitamin A supplementation compliance was high, and most children were vitamin A sufficient, hence daily egg consumption only marginally prevented a decrease in RBP concentration. This is similar to findings from the Mazira project in Malawi^53^ and the Lulun project in Ecuador^11^ where the provision of daily eggs in a population with low VAD did not affect vitamin A status.

Our study had some limitations. Mothers were not directly observed for hygienic practices and egg preparation methods; however, this was partly mitigated by health education on hygiene and food preparation provided to mothers at study enrolment and during field workers’ weekly visits. In addition, we did not collect stool samples to analyse the causative organisms for the diarrhoea, nor tested the eggs for bacterial contamination. However, this contamination was minimised by using a sole reputable supplier for the entire duration of the study. The study was also not blinded so we did not have a ‘true’ control group (placebo) which might have resulted in reporting bias. Lastly, the morbidity and dietary intake data and egg intake estimation were based on self-reports which may cause recall and social desirability bias.

Notwithstanding, the strengths of the study included a secondary outcome of an RCT, the risk of potential confounders was minimised, a large sample size powered to detect small effects, and the qualitative component to explore perceptions of mothers about gastrointestinal morbidity in the context of egg consumption.

In conclusion, complementary feeding with one egg per day for 6 months did not reduce the incidence and duration of morbidity symptoms among 6-< 9 months infants from a low socioeconomic community in South Africa. However, it increased the incidence of gastrointestinal morbidity by 40% and had no effect on respiratory morbidity. Careful attention should be paid during the implementation of complementary feeding interventions for example with eggs, to reduce the burden of enteric infections through appropriate hygiene practices.

## Electronic supplementary material

Below is the link to the electronic supplementary material.

Supplementary Material 1

## Data Availability

The datasets used and/or analysed during the current study are available from the corresponding author upon reasonable request, upon approvals from the relevant authorities.
