# Supplementary material for: Gastrointestinal and respiratory morbidity when introducing eggs as complementary food: a randomised controlled trial in South African infants
Source: Sci Rep. 2024 Oct 29;14:25881. doi: 10.1038/s41598-024-76169-4 (PMC11519461; doi:10.1038/s41598-024-76169-4)
Supplement: Supplementary file 1 — Supplementary Material 1 [file 41598_2024_76169_MOESM1_ESM.pdf]

Supplementary information

Gastrointestinal and respiratory morbidity when introducing eggs as complementary food: A randomised controlled trial in South African infants

Regina Nakiranda[1*], Linda Malan[1], Hannah Ricci[1,2], Herculina S Kruger[1], Arista Nienaber[1], Marina Visser[1], Cristian Ricci[2], Mieke Faber[1,3,] Cornelius M. Smuts[1]

[1]North-West University (Centre of Excellence for Nutrition), Potchefstroom, South Africa.

[2]North-West University (Africa Unit for Transdisciplinary Health Research (AUTHeR), Potchefstroom, South Africa.

[3]South African Medical Research Council (Non-Communicable Diseases Research Unit), Tygerberg, South Africa.

*Corresponding author: Regina Nakiranda, Email address: reginaki30@gmail.com ORCID:0000-0001-8057-2751

Supplementary Table S1: Estimated weekly egg intake of intervention and control group during the 6-month study

	Intervention group (n = 250)	Control group (n = 250)	P-value
Mean weekly egg intake ± SD	6.5 ± 1.4	1.14 ± 1.0	<0.001
Median weekly intake (5th, 95th percentile)	7.0 (4.0, 7.0)	0.5 (0.0, 5.0)	<0.001

Supplementary Table S2. Foods consumed during the week preceding baseline, midpoint and endpoint, expressed as a percentage of babies for whom the food frequency questionnaire was completed.

Food group	Food	Days per week	Group	Baseline, n (%)	Midpoint n (%)	Endpoint n (%)
Milk feeds	Breast milk	Every day	Intervention	151 (60.4)	127 (54.5)	112 (48.7)
			Control	157 (62.8)	127 (55.9)	112 (51.9)
			p-value	*0.581*	*0.756*	*0.505*
	Formula milk	Every day	Intervention	127 (50.8)	109 (46.8)	84 (36.5)
			Control	123 (49.2)	93 (41.0)	73 (33.8)
			p-value	*0.721*	*0.788*	*0.721*
Dairy	Milk	≥ 4 days	Intervention	9 (3.6)	46 (19.7)	73 (31.7)
			Control	16 (6.4)	44 (19.4)	48 (22.2)
			p-value	*0.151*	*0.923*	***0.024***
	Yoghurt	≥ 4 days	Intervention	3 (1.2)	18 (7.7)	30 (13.0)
			Control	8 (3.2)	20 (8.8)	33 (15.3)
			p-value	*0.127*	*0.673*	*0.498*
Baby foods	Pureed baby foods	≥ 4 days	Intervention	53 (21.2)	44 (18.0)	30 (13.0)
			Control	54 (21.6)	47 (20.7)	30 (13.9)
			p-value	*0.913*	*0.624*	*0.794*
	Infant cereal	≥ 4 days	Intervention	161 (64.4)	95 (40.8)	56 (24.3)
			Control	146 (58.4)	75 (33.0)	36 (16.7)
			p-value	*0.168*	*0.086*	***0.045***
Cereals, roots, tubers	Instant maize porridge	≥ 4 days	Intervention	26 (10.4)	43 (18.5)	52 (22.6)
			Control	28 (11.2)	34 (15.0)	50 (23.1)
			p-value	*0.773*	*0.318*	*0.892*
	Maize meal	≥ 4 days	Intervention	47 (18.8)	116 (49.8)	146 (63.5)
			Control	51 (20.4)	107 (47.1)	151 (69.9)
			p-value	*0.652*	*0.570*	*0.150*
	Porridge, other than maize	≥ 4 days	Intervention	15 (6.0)	32 (13.7)	40 (17.4)
			Control	18 (7.2)	42 (18.5)	36 (16.7)
			p-value	*0.589*	*0.164*	*0.839*
	Breakfast cereal	≥ 4 days	Intervention	16 (6.4)	34 (14.6)	52 (22.6)
			Control	12 (4.8)	41 (18.1)	40 (18.5)
			p-value	*0.437*	*0.314*	*0.268*
	Potato	≥ 4 days	Intervention	15 (6.0)	41 (17.6)	22 (9.6)
			Control	37 (14.8)	35 (15.4)	34 (15.7)
			p-value	***0.001***	*0.529*	***0.049***
Animal source foods	Chicken	≥ 1 day	Intervention	63 (25.2)	134 (57.5)	158 (68.7)
			Control	71 (28.4)	139 (61.2)	152 (70.4)
			p-value	*0.419*	*0.416*	*0.701*
	Meat	≥ 1 day	Intervention	13 (5.2)	35 (15.0)	65 (28.3)
			Control	11 (4.4)	51 (22.5)	84 (39.9)
			p-value	*0.676*	***0.041***	***0.017***
	Liver	≥ 1 day	Intervention	38 (15.2)	64 (27.5)	72 (31.3)
			Control	40 (16.0)	73 (32.2)	70 (32.4)
			p-value	*0.805*	*0.271*	*0.803*
	Fish	≥ 1 day	Intervention	11 (4.4)	41 (17.6)	64 (27.8)
			Control	10 (4.0)	33 (14.5)	60 (27.8)
			p-value	*0.824*	*0.372*	*0.991*
	Egg	≥ 4 days	Intervention	12 (4.8)	205 (88.0)	189 (82.8)
			Control	13 (5.2)	26 (11.5)	29 (13.4)
			p-value	*0.829*	***<0.001***	***<0.001***

Baseline: Intervention group *n* = 250; Control group *n* = 250; Midpoint: Intervention group *n* = 233; Control group *n* = 227; Endpoint: Intervention group *n* = 230 control *n* = 216. Differences between groups assessed with Chi-Square tests.

Supplementary Table S3: Vitamin A status and supplementation before and after the 6-month egg intervention

	Intervention group	Control group	B-value	P-value[1]
**Retinol binding protein (**µmol/L), (mean ± SD)				
Baseline	1.795 ± 0.491	1.863 ± 0.552		0.170
Endpoint	1.779 ± 0.458	1.811 ± 0.518		0.492
Change	-0.016	-0.052	0.469 (0.393-0.544)	**<0.001**
**Vitamin A deficiency (RBP ≤0.7 µmol/L), [n (%)]**				
Baseline	0 (0.0)	0 (0.0)		N/A
End	0 (0.0)	0 (0.0)		N/A
**Vitamin A supplementation [n (%)]**				
Baseline	137 (54.8)	146 (58.4)		0.417
Endpoint	208 (83.2)	204 (81.6)		0.639

Baseline: Intervention group n=250; Control group n=250 ; Endpoint: Intervention group: n=230; Control group n=216

[1] Differences of retinol binding protein (RBP) between intervention and control groups were assessed by independent student t-tests. Effect of intervention was assessed by ANCOVA adjusting for baseline RBP, and vitamin A supplementation at endpoint. RBP was corrected for inflammation using the BRINDA method[1]. Difference in vitamin A supplementation between intervention and control groups was assessed by Chi Square tests. P <0.05 was considered significant

Reference

1 Namaste, S. M. & Rohner, F. Adjusting ferritin concentrations for inflammation: Biomarkers Reflecting Inflammation and Nutritional Determinants of Anemia (BRINDA) project. *Am J Clin Nutr* **106**, 359s-371s (2017). https://doi.org:10.3945/ajcn.116.141762
